# Supplementary material for: Feature screening for survival trait with application to TCGA high-dimensional genomic data
Source: PeerJ. 2022 Mar 10;10:e13098. doi: 10.7717/peerj.13098 (PMC8918142; doi:10.7717/peerj.13098)
Supplement: Supplemental Information 1 [file peerj-10-13098-s001.docx]

Web-based Supplementary Materials for

” **Feature Screening for Survival Trait with Application to TCGA High-dimensional Genomic Data**”

by

Jie-Huei Wang, Cai-Rong Li and Po-Lin Hou

**
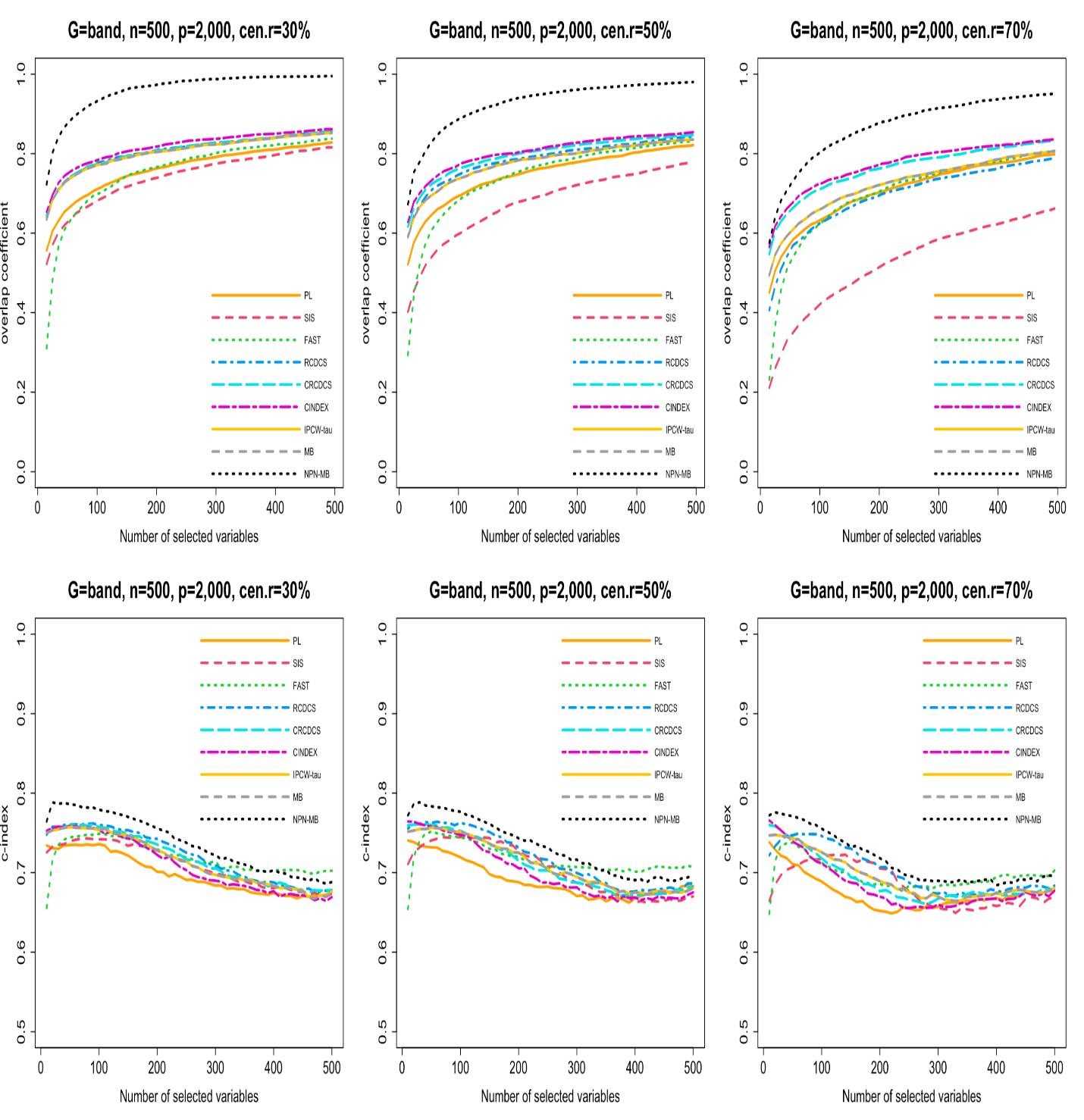
**

**Supplementary Figure 1: The multi-panel figure contains the mean number of overlap coefficient (top three panels)) and *c*-index (bottom three panels)) among 200 replications by the number of selected features for the simulation study 3 with band structure based on PH model.** The left, medium, and right plots are based on censoring rates of 30%, 50%, and 70%, respectively. A larger mean number of overlap coefficient indicates highly similarity with a ground truth set of predictors, and a larger c-index indicates better prediction accuracy. Note that the underlying survival model has 15 true predictors.


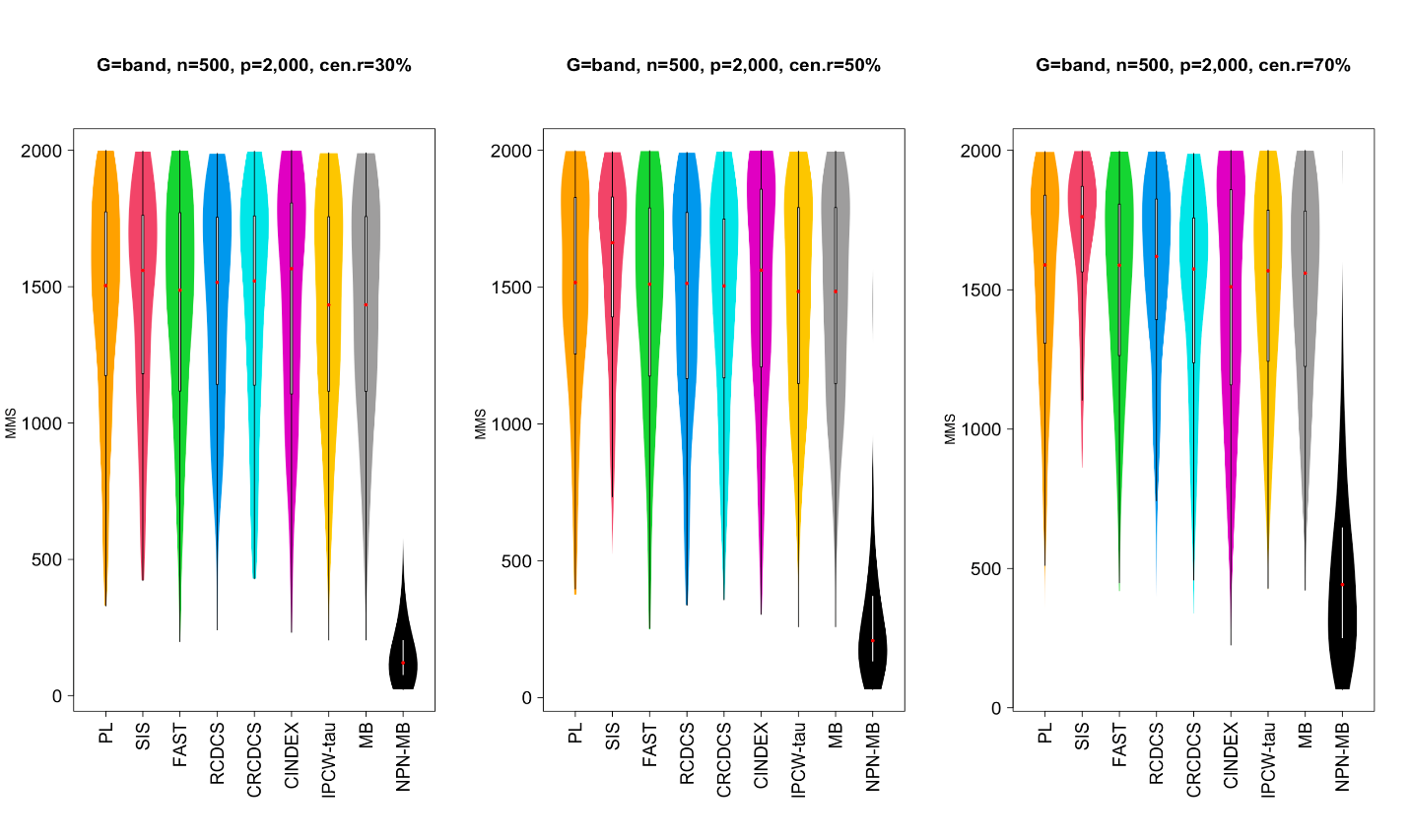


**Supplementary Figure 2: The violin chart of minimum of model size (MMS) measure among 200 replications for the simulation study 3 with band structure based on PH model.**

The left, medium, and right plots are based on censoring rates of 30%, 50%, and 70%, respectively. A smaller MMS value indicates the higher accuracy of feature screening.

**
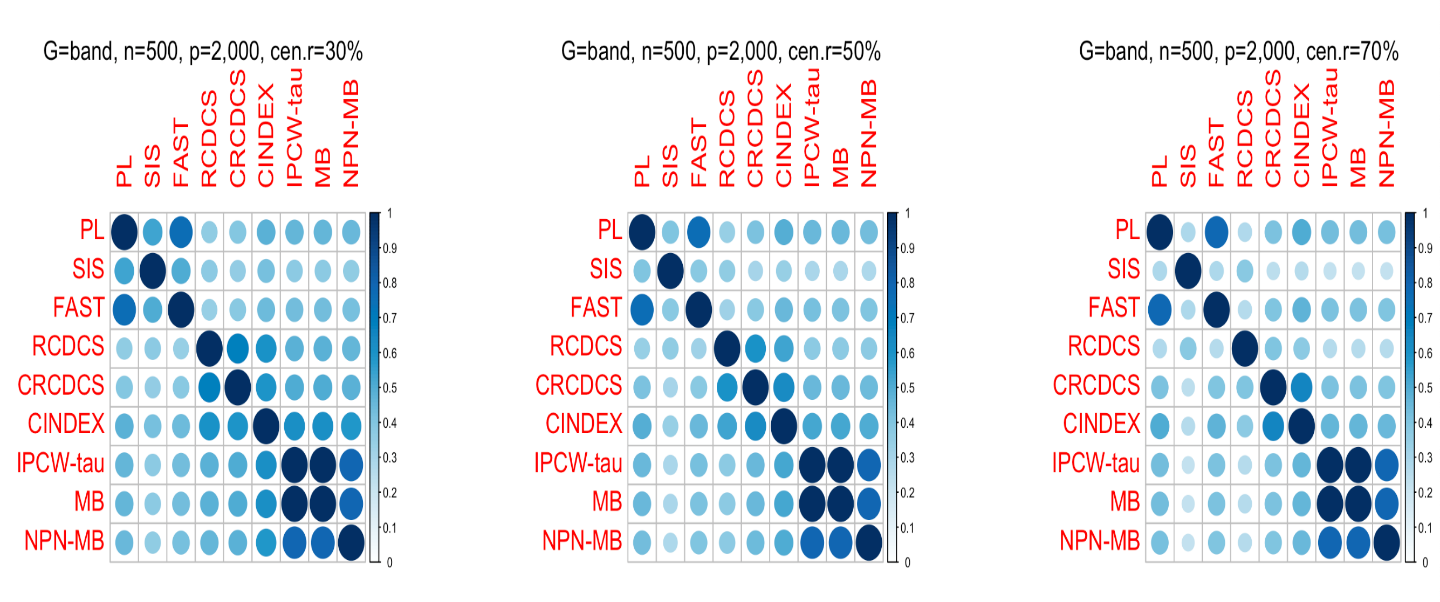
**

**Supplementary Figure 3: The average of Jaccard index among 200 replications for the simulation study 3 with band structure based on PH model.**

The left, medium, and right plots are based on censoring rates of 30%, 50%, and 70%, respectively.


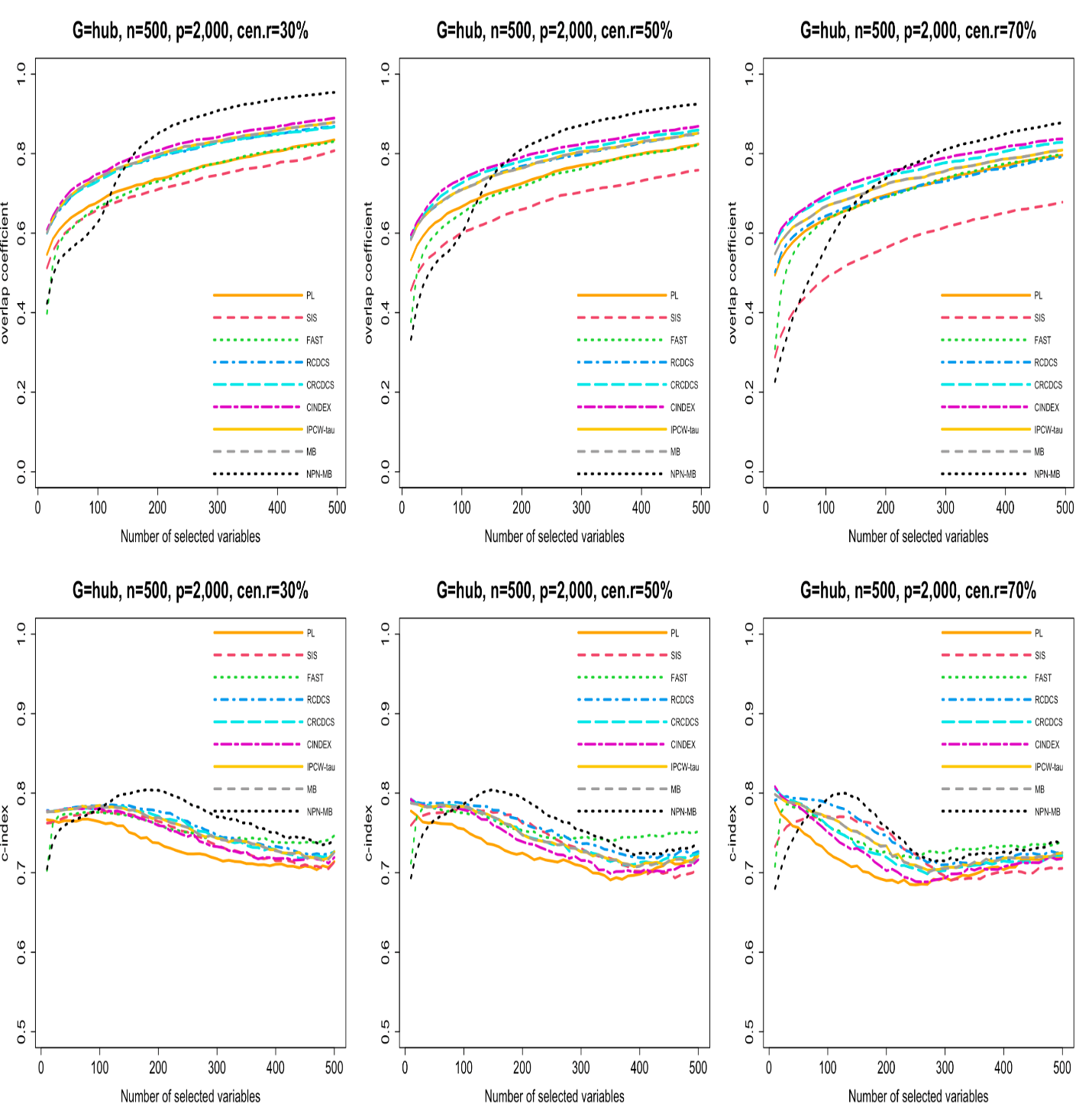


**Supplementary Figure 4: The multi-panel figure contains the mean number of overlap coefficient (top three panels)) and *c*-index (bottom three panels)) among 200 replications by the number of selected features for the simulation study 3 with hub structure based on PH model.** The left, medium, and right plots are based on censoring rates of 30%, 50%, and 70%, respectively. A larger mean number of overlap coefficient indicates highly similarity with a ground truth set of predictors, and a larger c-index indicates better prediction accuracy. Note that the underlying survival model has 15 true predictors.


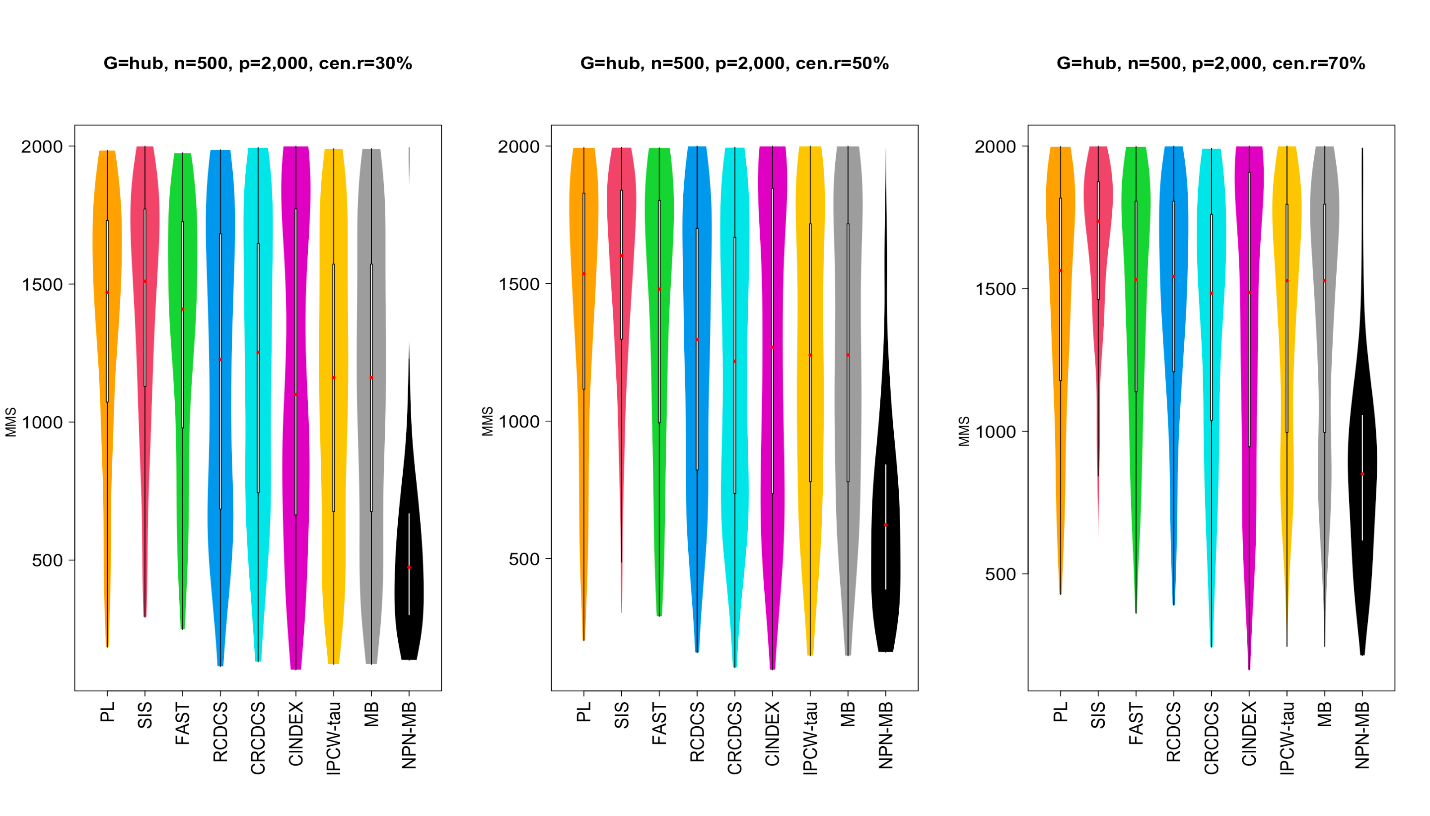


**Supplementary Figure 5: The violin chart of minimum of model size (MMS) measure among 200 replications for the simulation study 3 with hub structure based on PH model.**

The left, medium, and right plots are based on censoring rates of 30%, 50%, and 70%, respectively. A smaller MMS value indicates the higher accuracy of feature screening.


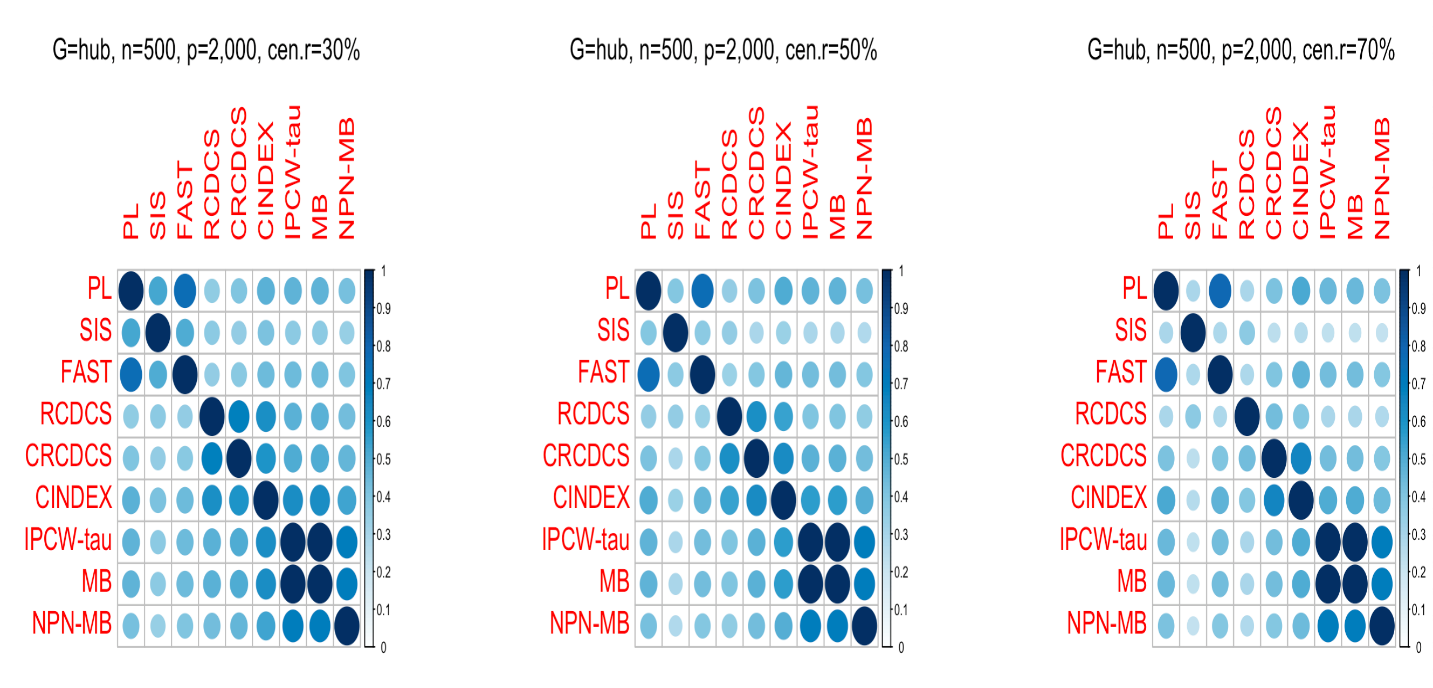


**Supplementary Figure 6: The average of Jaccard index among 200 replications for the simulation study 3 with hub structure based on PH model.**

The left, medium, and right plots are based on censoring rates of 30%, 50%, and 70%, respectively.


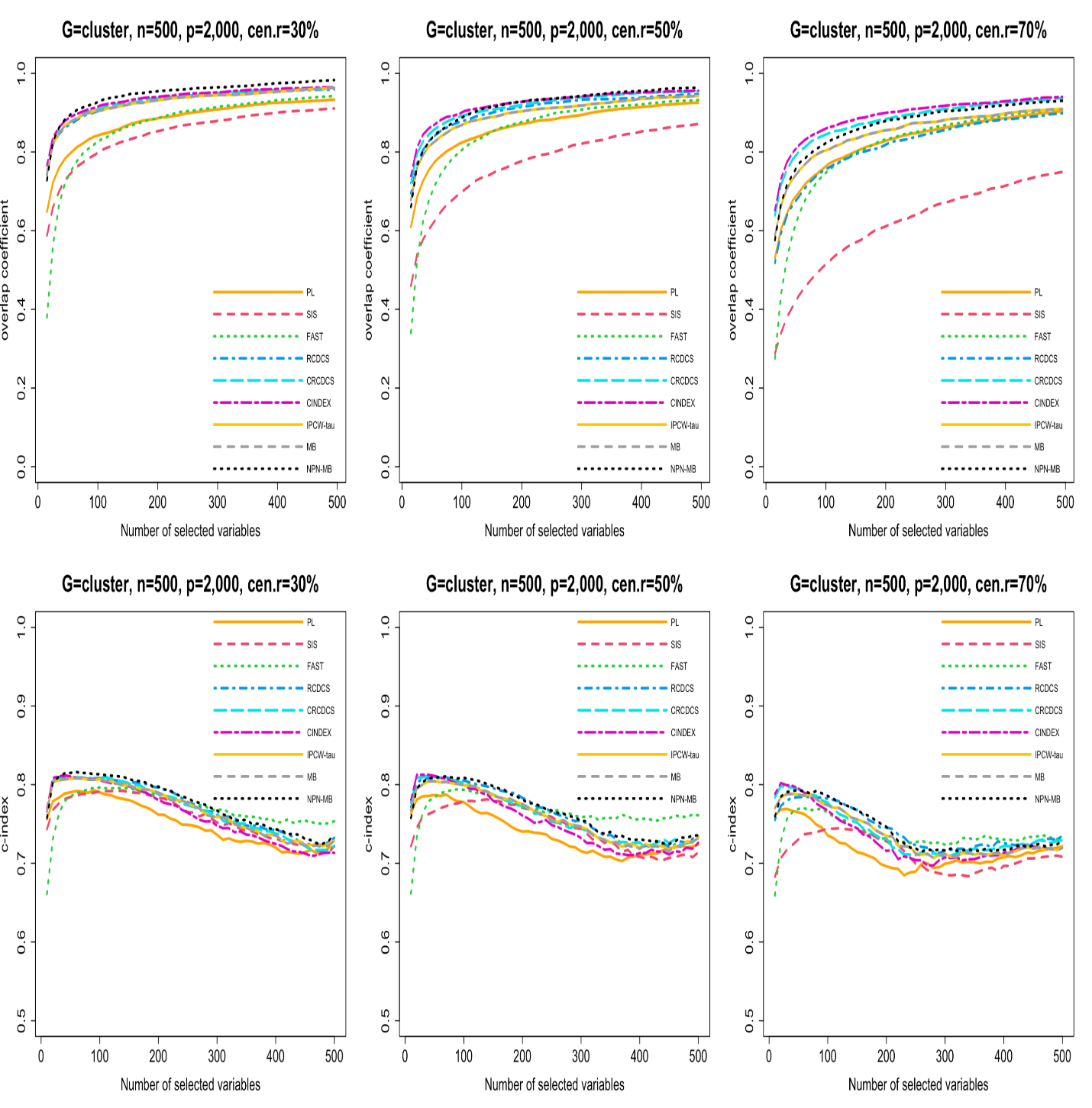


**Supplementary Figure 7: The multi-panel figure contains the mean number of overlap coefficient (top three panels)) and *c*-index (bottom three panels)) among 200 replications by the number of selected features for the simulation study 3 with cluster structure based on PH model.** The left, medium, and right plots are based on censoring rates of 30%, 50%, and 70%, respectively. A larger mean number of overlap coefficient indicates highly similarity with a ground truth set of predictors, and a larger c-index indicates better prediction accuracy. Note that the underlying survival model has 15 true predictors.


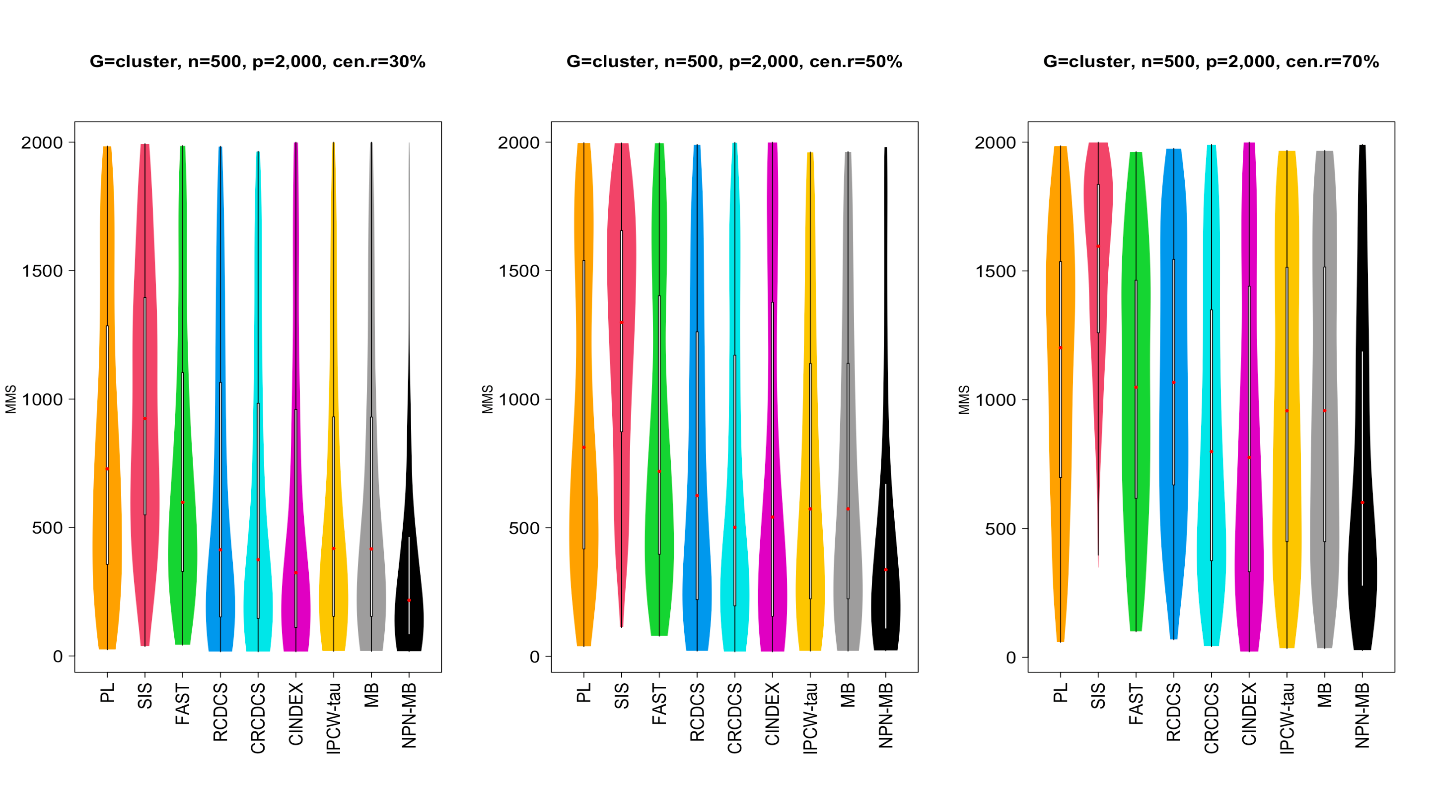


**Supplementary Figure 8: The violin chart of minimum of model size (MMS) measure among 200 replications for the simulation study 3 with cluster structure based on PH model.**

The left, medium, and right plots are based on censoring rates of 30%, 50%, and 70%, respectively. A smaller MMS value indicates the higher accuracy of feature screening.


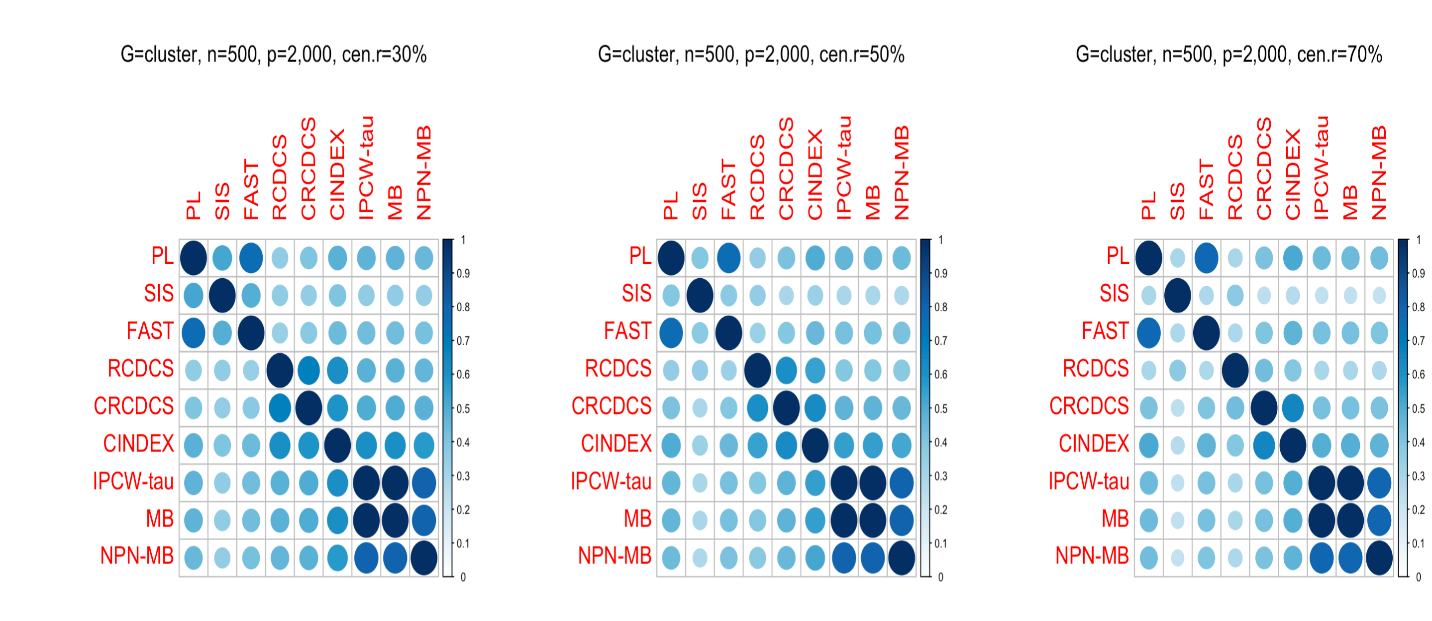


**Supplementary Figure 9: The average of Jaccard index among 200 replications for the simulation study 3 with cluster structure based on PH model.**

The left, medium, and right plots are based on censoring rates of 30%, 50%, and 70%, respectively.


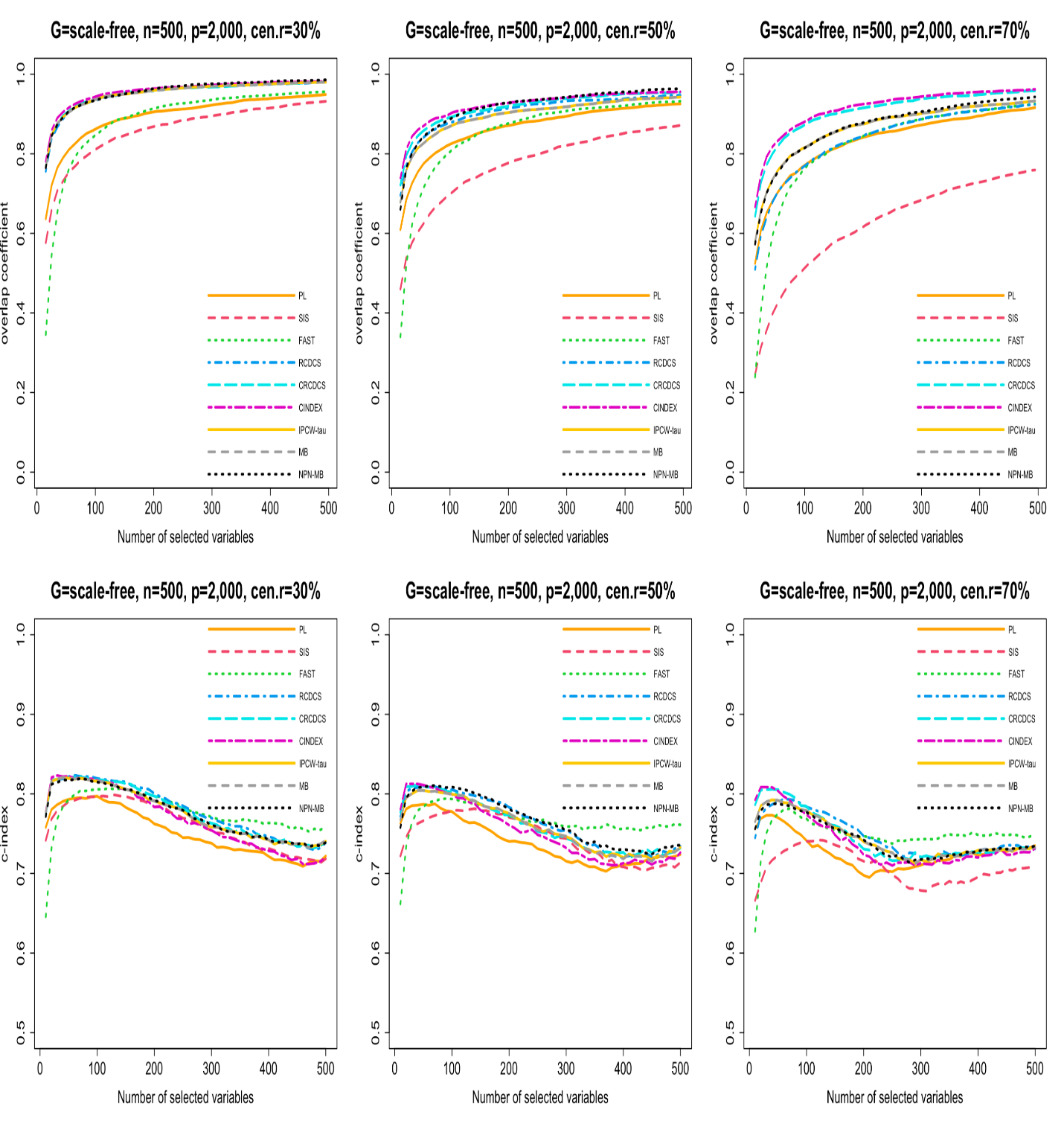


**Supplementary Figure 10: The multi-panel figure contains the mean number of overlap coefficient (top three panels)) and *c*-index (bottom three panels)) among 200 replications by the number of selected features for the simulation study 3 with scale-free structure based on PH model.** The left, medium, and right plots are based on censoring rates of 30%, 50%, and 70%, respectively. A larger mean number of overlap coefficient indicates highly similarity with a ground truth set of predictors, and a larger c-index indicates better prediction accuracy. Note that the underlying survival model has 15 true predictors.


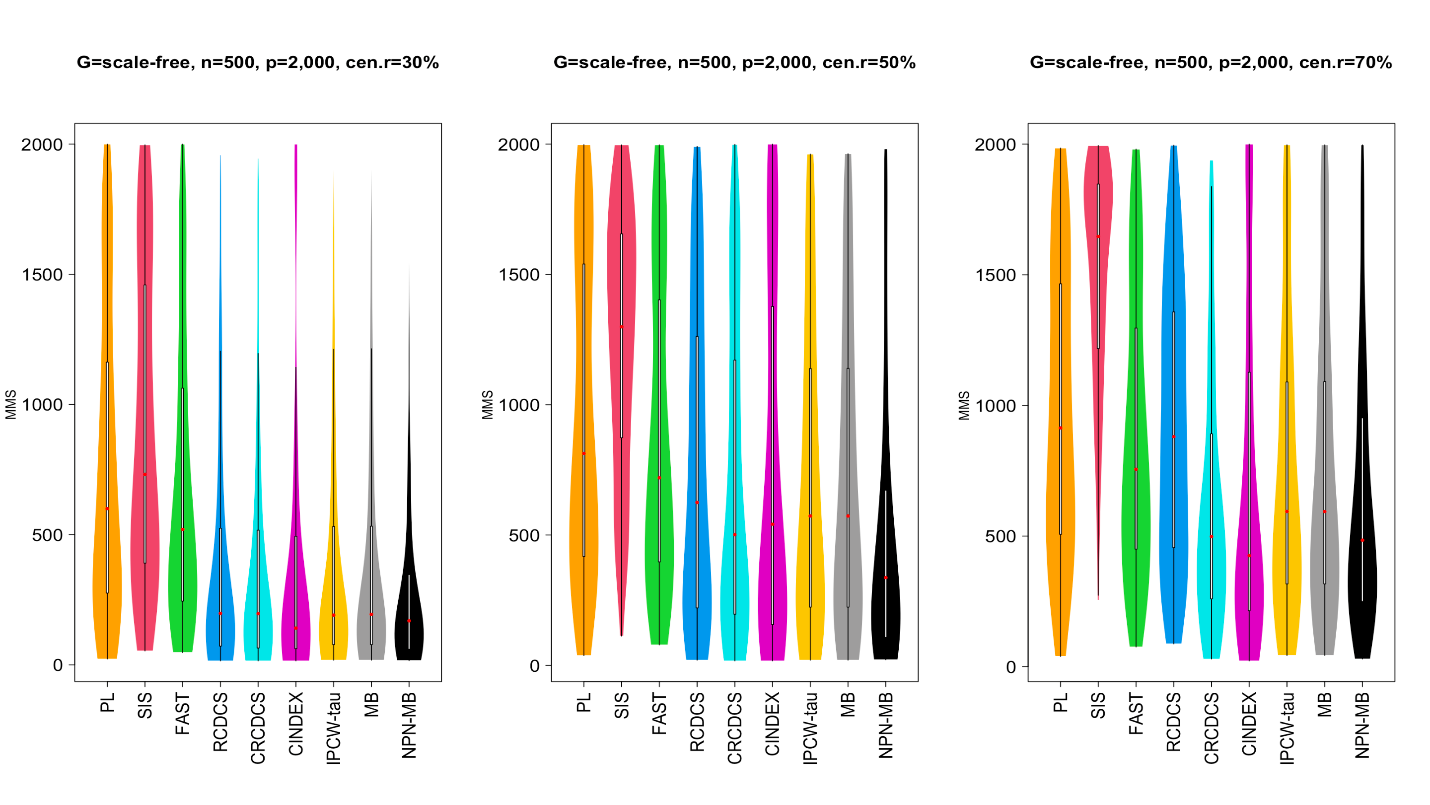


**Supplementary Figure 11: The violin chart of minimum of model size (MMS) measure among 200 replications for the simulation study 3 with scale-free structure based on PH model.**

The left, medium, and right plots are based on censoring rates of 30%, 50%, and 70%, respectively. A smaller MMS value indicates the higher accuracy of feature screening.


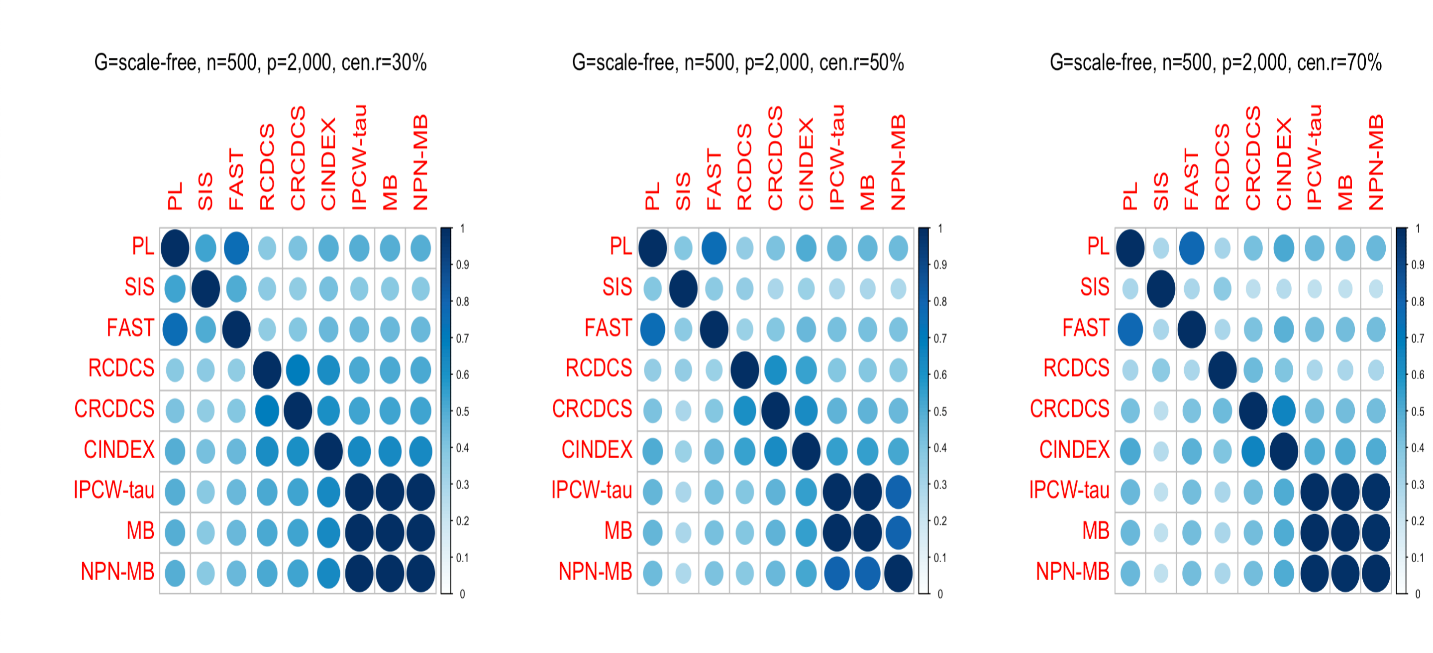


**Supplementary Figure 12: The average of Jaccard index among 200 replications for the simulation study 3 with scale-free structure based on PH model.**

The left, medium, and right plots are based on censoring rates of 30%, 50%, and 70%, respectively.

**The Cancer Genome Atlas** **head and neck squamous cell carcinoma (HNSCC) data**

The TCGA HNSCC RNA-Seq expression data which were collected using the IlluminaHiseq RNAseq V2 platform, together with the phenotype data containing the survival time and censoring status data, can be downloaded from the R package ‘GEInter’.

Data are available on 484 subjects. The response is overall survival, which is subject to right censoring. The censoring rate is about 58%. A total of 18,409 gene expression measurements are available. As the number of disease-associated biomarkers is not expected to be large, we conduct prescreening using marginal Cox models, which can also improve stability. The top 2,000 genes with the smallest p-values are selected for downstream analysis. We take five random splits of the whole data into 387:97 training/test sets of the data to evaluate the performance of all methods for survival prediction in the TCGA HNSCC data.

We apply eight screening methods, “PL”, “SIS”, “FAST”, “RCDCS”, “CRCDCS”, “CINDEX” “IPCW-tau”, “IPCW-tau (NPN-MB)”, to the TCGA HNSCC data. After grid search from the top 10 to the top 300 ranked genes, the best overall prediction performance of all methods is attained by using the top 170 genes, so the top-ranked 170 predictors are selected as the candidate covariates for each method, and the Cox’s regression model with the candidate covariates and the MCP penalty is applied to the training data to establish the final prediction model. Besides, the MCP-penalized Cox model with the top 2,000 genes selected by the univariate Cox’s test is applied to the training data to build the prediction model. We also take the published biomarker genes (*GIMAP6, SELL, TIFAB, KCNA3, CCR4*) related to HNSCC (Ran et al., 2021) as a survival prediction model to make comparisons.

The prediction accuracy performances for different methods are evaluated and the numerical results are provided in Table S1 that reports the median of the survival prediction results among five folds. We can see that the proposed IPCW-tau (NPN-MB) method outperforms the alternative methods for survival prediction in the TCGA HNSCC test data.

In addition, we apply the proposed IPCW-tau (NPN-MB) method for whole data to identify several important biomarker genes and estimate the correspondence parameters by penalized Cox’s regression model with the MCP penalty. Please see Table S2 for the list of selected associated predictors. We identify nine genes and find the four genes (*PITPNM3*, *MXD4*, *ABCB1*, *BATF*) that have been shown to be related to HNSCC in the literatures (Aravind et al., 2021, Wu et al., 2019, da Silva et al., 2021, Duz et al., 2021, Wang et al., 2020, and Wen et al., 2015).

**Supplementary Table 1:**

**Results (median of prediction accuracy of different methods in the TCGA HNSCC data over 5 random splits of 387:97 training /test sets); all feature screening methods and a published biomarker genes model are applied together with the MCP penalized Cox regression.**

|  | PL | SIS | FAST | RCDCS | CRCDCS | CINDEX | IPCW  -tau | NPN  -MB | Ordinary  -MCP | Ran et al.  (2021) |
| --- | --- | --- | --- | --- | --- | --- | --- | --- | --- | --- |
| Deviance | 6.8319 | 0.7690 | 6.3660 | -1.4770 | 0.7243 | 5.8908 | 2.0246 | -3.1675 | 276.269 | -1.0546 |
| c-index | 0.5842 | 0.6083 | 0.5818 | 0.5985 | 0.6130 | 0.5988 | 0.6053 | 0.6160 | 0.6215 | 0.5457 |
| NOSF | 12 | 10 | 13 | 11 | 16 | 13 | 13 | 9 | 44 | 1 |

**Supplementary Table 2:**

**Selected genes with their correspondence estimate by IPCW-tau (NPN-NB) screening procedure with MCP penalty for the whole TCGA HNSCC data.**

| gene | estimate | Citation |
| --- | --- | --- |
| *ZNF266* | -0.46563237 |  |
| *SEC11A* | 0.20228840 |  |
| *PITPNM3* | -0.15584711 | Aravind et al. (2021) |
| *MXD4* | -0.13870271 | Wu et al. (2019) |
| *ABCB1* | -0.01950197 | da Silva et al. (2021),  Duz et al. (2021), and  Wang et al. (2020) |
| *MAST4* | -0.23504445 |  |
| *SERINC3* | 0.04911169 |  |
| *BATF* | -0.27027155 | Wen et al. (2015) |
| *PITPNB* | 0.03290633 |  |

**The Cancer Genome Atlas** **lung adenocarcinoma (LUAD) data**

After excluding patients with missing survival time data, our analysis is focused on the subset of the TCGA LUAD data with 505 patients and 20,501 gene expression variables. The censoring rate in the data is about 64%. Due to the number of disease-associated biomarkers is not expected to be large, the top 2,000 genes with the smallest p-values based on marginal Cox’s model are selected for downstream analysis. We take five random splits of the whole data into 404:101 training/test sets of the data to evaluate the performance of all methods for survival prediction in the TCGA LUAD data.

We apply eight screening methods, “PL”, “SIS”, “FAST”, “RCDCS”, “CRCDCS”, “CINDEX” “IPCW-tau”, “IPCW-tau (NPN-MB)”, to the TCGA LUAD data. After grid search from the top 10 to the top 300 ranked genes, the best overall prediction performance of all methods is attained by using the top 140 genes, so the top-ranked 140 predictors are selected as the candidate covariates for each method, and the Cox’s regression model with the candidate covariates and the MCP penalty is applied to the training data to establish the final prediction model. Besides, the MCP-penalized Cox model with the top 2,000 genes selected by the univariate Cox’s test is applied to the training data to build the prediction model. We also take the published biomarker genes (*ALK, BRAF, EGFR, ROS1*) related to LUAD (Chen et al., 2021) as a survival prediction model to make comparisons.

The prediction accuracy performances for different methods are evaluated and the numerical results are provided in Table S3 that reports the median of the survival prediction results among five folds. We can see that the proposed IPCW-tau (NPN-MB) method outperforms the alternative methods for survival prediction in the TCGA LUAD test data.

In addition, we apply the proposed IPCW-tau (NPN-MB) method for whole data to identify several important biomarker genes and estimate the correspondence parameters by penalized Cox’s regression model with the MCP penalty. Please see Table S4 for the list of selected associated predictors. We identify fourteen genes and find the seven genes (*EPB41L5*, *INPP5J*, *KRT16*, *MS4A1*, *MYLIP*, *PEBP1*, *SFTPB*) that have been shown to be related to LUAD in the literatures (Li et al., 2020, Zhang et al., 2020, Yuanhua et al., 2019, Song et al., 2020, Liu et al., 2021, Li et al., 2020, Zhang et al., 2021, Cao et al., 2021, and Zhang et al., 2019).

**Supplementary Table 3:**

**Results (median of prediction accuracy of different methods in the TCGA LUAD data over 5 random splits of 404:101 training /test sets); all feature screening methods and a published biomarker genes model are applied together with the MCP penalized Cox regression.**

|  | PL | SIS | FAST | RCDCS | CRCDCS | CINDEX | IPCW  -tau | NPN  -MB | Ordinary-MCP | Chen et al.  (2021) |
| --- | --- | --- | --- | --- | --- | --- | --- | --- | --- | --- |
| Deviance | 7.7639 | 19.8600 | 12.79617 | 2.8480 | 3.8522 | -1.3321 | -1.2874 | -3.4570 | 1701.964 | -0.9546 |
| c-index | 0.5873 | 0.6083 | 0.6257 | 0.6463 | 0.6400 | 0.6302 | 0.6421 | 0.6527 | 0.5962 | 0.5821 |
| NOSF | 14 | 17 | 16 | 18 | 14 | 11 | 16 | 11 | 83 | 1 |

**Supplementary Table 4:**

**Selected genes with their correspondence estimate by IPCW-tau (NPN-NB) screening procedure with MCP penalty for the whole TCGA LUAD data.**

| gene | estimate | Citation |
| --- | --- | --- |
| ADH1B | 0. 1015 |  |
| C1QTNF6 | 0. 3162 |  |
| CDCP1 | 0. 0815 |  |
| CNIH | 0. 5212 |  |
| EPB41L5 | -0. 1287 | Li et al. (2020) |
| EPS8L3 | 0.0087 |  |
| INPP5J | -0.1429 | Zhang et al. (2020) |
| KRT16 | -0.1192 | Yuanhua et al. (2019) |
| MS4A1 | -0.1302 | Song et al. (2020) |
| MYLIP | -0.4459 | Liu et al. (2021), and  Li et al. (2020) |
| PEBP1 | -0.4761 | Zhang et al. (2021) |
| PLEC | 0.1959 |  |
| RCBTB2 | -0.3733 |  |
| SELENBP1 | 0.2768 |  |
| SFTPB | -0.0878 | Cao et al. (2021), and  Zhang et al. (2019) |

**The Cancer Genome Atlas** **breast invasive carcinoma (BRCA) data**

After excluding patients with missing survival time data, our analysis is focused on the subset of the TCGA BRCA data with 1,094 patients and 20,501 gene expression variables. The censoring rate in the data is about 86%. Due to the number of disease-associated biomarkers is not expected to be large, the top 2,000 genes with the smallest p-values based on marginal Cox’s model are selected for downstream analysis. We take five random splits of the whole data into 876:218 training/test sets of the data to evaluate the performance of all methods for survival prediction in the TCGA BRCA data.

We apply eight screening methods, “PL”, “SIS”, “FAST”, “RCDCS”, “CRCDCS”, “CINDEX” “IPCW-tau”, “IPCW-tau (NPN-MB)”, to the TCGA LUAD data. After grid search from the top 10 to the top 300 ranked genes, the best overall prediction performance of all methods is attained by using the top 210 genes, so the top-ranked 210 predictors are selected as the candidate covariates for each method, and the Cox’s regression model with the candidate covariates and the MCP penalty is applied to the training data to establish the final prediction model. Besides, the MCP-penalized Cox model with the top 2,000 genes selected by the univariate Cox’s test is applied to the training data to build the prediction model. We also take the published biomarker genes (*TMEM190, TUBA3D, LYVE1, LILBR5, CD209*) related to BRCA (Liu et al., 2019) as a survival prediction model to make comparisons.

The prediction accuracy performances for different methods are evaluated and the numerical results are provided in Table S5 that reports the median of the survival prediction results among five folds. We can see that the proposed IPCW-tau (NPN-MB) method outperforms the alternative methods for survival prediction in the TCGA BRCA test data.

In addition, we apply the proposed IPCW-tau (NPN-MB) method for whole data to identify several important biomarker genes and estimate the correspondence parameters by penalized Cox’s regression model with the MCP penalty. Please see Table S6 for the list of selected associated predictors. We identify ten genes and find the four genes (*EDA2R*, *PCMT1*, *QPRT*, *SKP1*) that have been shown to be related to BRCA in the literatures (Liu, Kain & Wang, 2012, Kyritsis et al. 2021, Liu et al. 2021, and Tian et al. 2020).

**Supplementary Table 5:**

**Results (median of prediction accuracy of different methods in the TCGA BRCA data over 5 random splits of 876:218 training /test sets); all feature screening methods and a published biomarker genes model are applied together with the MCP penalized Cox regression.**

|  | PL | SIS | FAST | RCDCS | CRCDCS | CINDEX | IPCW  -tau | NPN  -MB | Ordinary  -MCP | Liu et al.  (2019) |
| --- | --- | --- | --- | --- | --- | --- | --- | --- | --- | --- |
| Deviance | 0.6100 | 0.5991 | 0.6847 | 0.6224 | 0.6041 | 0.6361 | 0.5432 | 0.6479 | 0.7192 | 0.5795 |
| *c*-index | 46.3608 | 381.8566 | 18.3825 | 76.5045 | 26.1614 | 53.1598 | 14.5303 | 1.7584 | 2015.243 | 0.5637 |
| NOSF | 28 | 88 | 22 | 29 | 21 | 28 | 21 | 15 | 78 | 2 |

**Supplementary Table 6:**

**Selected genes with their correspondence estimate by IPCW-tau (NPN-NB) screening procedure with MCP penalty for the whole TCGA BRCA data.**

| gene | estimate | Citation |
| --- | --- | --- |
| *C21orf57* | -0.30371804 |  |
| *EDA2R* | 0.09746804 | Liu, Kain & Wang (2012) |
| *JRKL* | 0.45789616 |  |
| *PCMT1* | 0.65613403 | Kyritsis et al. (2021) |
| *QPRT* | 0.13559978 | Liu et al. (2021) |
| *ROPN1L* | -0.25100467 |  |
| *SKP1* | 0.70641965 | Tian et al. (2020) |
| *SPINK8* | 0.05213272 |  |
| *TANK* | -0.97414124 |  |
| *ZNF674* | 0.20787107 |  |

**REFERENCES**

**Aravind A, Palollathil A, Rex D, Kumar K, Vijayakumar M, Shetty R, Codi J, Prasad T, Raju R. 2021.** A multi-cellular molecular signaling and functional network map of C-C motif chemokine ligand 18 (CCL18): a chemokine with immunosuppressive and pro-tumor functions. *Journal of cell communication and signaling* [DOI 10.1007/s12079-021-00633-3](https://doi.org/10.1007/s12079-021-00633-3).

**da Silva G, de Matos LL, Kowalski LP, Kulcsar M, Leopoldino AM. 2021.** Profile of sphingolipid-related genes and its association with prognosis highlights sphingolipid metabolism in oral cancer. *Cancer biomarkers : section A of Disease markers* **32**:49–63 [DOI 10.3233/CBM-203100](https://doi.org/10.3233/CBM-203100).

**Cao B, Wang P, Gu L, Liu J. 2021.** Use of four genes in exosomes as biomarkers for the identification of lung adenocarcinoma and lung squamous cell carcinoma. *Oncology letters* **21**:249

[DOI 10.3892/ol.2021.12510](https://doi.org/10.3892/ol.2021.12510).

**Chen L, Zeng H, Xiang Y, Huang Y, Luo Y, Ma X. 2021.** Histopathological Images and. Multi-Omics Integration Predict Molecular Characteristics and Survival in Lung Adenocarcinoma. *Frontiers in cell and developmental biology* **9**:720110 DOI 10.3389/fcell.2021.720110.

**Duz MB, Karatas OF. 2021.** Differential expression of ABCB1, ABCG2, and KLF4 as. putative indicators for paclitaxel resistance in human epithelial type 2 cells. *Molecular biology reports***48:**1393–1400 [DOI 10.1007/s11033-021-06167-6](https://doi.org/10.1007/s11033-021-06167-6).

**Kyritsis KA, Akrivou MG, Giassafaki LN, Grigoriadis NG, Vizirianakis IS. 2021.** Analysis of TCGA data of. differentially expressed EMT‑related genes and miRNAs across various malignancies to identify potential biomarkers. *World Academy of Sciences Journal* **3**:6. [DOI 10.3892/wasj.2020.77](https://doi.org/10.3892/wasj.2020.77).

**Li J, Li Z, Zhao S, Song,Y, Si L, Wang X. 2020.** Identification key genes, key miRNAs and key transcription factors of lung adenocarcinoma. *Journal of thoracic disease***12**:1917–1933

[DOI 10.21037/jtd-19-4168](https://doi.org/10.21037/jtd-19-4168).

**Li H, Tong L, Tao H, Liu Z. 2020.** Genome-wide analysis of the hypoxia-related DNA methylation-driven genes in lung adenocarcinoma progression. *Bioscience reports***40**

[DOI 10.1042/BSR20194200](https://doi.org/10.1042/BSR20194200).

**Liu CL, Cheng SP, Chen MJ, Lin CH, Chen SN, Kuo YH, Chang,YC. 2021.** Quinolinate. Phosphoribosyltransferase Promotes Invasiveness of Breast Cancer Through Myosin Light Chain Phosphorylation. *Frontiers in endocrinology***11**:621944. [DOI 10.3389/fendo.2020.621944](https://doi.org/10.3389/fendo.2020.621944).

**Liu R, Kain M, Wang L. 2012.** Inactivation of X-linked tumor suppressor genes in human cancer. *Future oncology (London, England)* **8:463–**481. [DOI 10.2217/fon.12.26](https://doi.org/10.2217/fon.12.26).

**Liu T, Yang C, Wang W, Liu C. 2021.** LncRNA SGMS1-AS1 regulates lung adenocarcinoma cell proliferation, migration, invasion, and EMT progression via miR-106a-5p/MYLI9 axis. *Thoracic cancer***12**:2104–2112 [DOI 10.1111/1759-7714.14043](https://doi.org/10.1111/1759-7714.14043).

**Liu L, Chen Z, Shi W, Liu H, Pang W. 2019.** Breast cancer survival prediction using seven prognostic biomarker genes. *Oncology letters* **18(3)**:2907–2916. [DOI 10.3892/ol.2019.10635](https://doi.org/10.3892/ol.2019.10635).

**Ran QC, Long SR, Ye Y, Xie C, XuXiao ZL, Liu YS, Pang HX, Sunchuri D, Teng NC, Guo ZL. 2021.** Mining TCGA database for prognostic genes in head and neck squamous cell carcinoma microenvironment. *Journal of dental sciences* **16(2)**:661–667 [DOI 10.1016/j.jds.2020.09.017](https://doi.org/10.1016/j.jds.2020.09.017).

**Song C, Guo Z, Yu D, Wang Y, Wang Q, Dong Z, Hu W. 2020.** A Prognostic Nomogram Combining Immune-Related Gene Signature and Clinical Factors Predicts Survival in Patients With Lung Adenocarcinoma. *Frontiers in oncology***10**:1300 [DOI 10.3389/fonc.2020.01300](https://doi.org/10.3389/fonc.2020.01300).

**Tian Z, He W, Tang J, Liao X, Yang Q, Wu Y, Wu G. 2020.** Identification of Important Modules and Biomarkers in. Breast Cancer Based on WGCNA. *OncoTargets and therapy* **13**:6805–6817.

[DOI 10.2147/OTT.S258439](https://doi.org/10.2147/OTT.S258439).

**Wang Y, Xu Y, Hua Q, Jiang Y, Liu P, Zhang W, Xiang R. 2020. N**ovel Prognostic Model. Based on Immune Signature for Head and Neck Squamous Cell Carcinoma. *BioMed research international***2020**:4725314 DOI [10.1155/2020/4725314](https://doi.org/10.1155/2020/4725314).

**Wen H, Tang J, Liu B, Sun C. 2015.** The expression and clinical significance of BATF2 in oral

tongue squamous cell carcinoma. *Chinese journal of stomatology* **50**:13–17.

**Wu Y, Wang Y, Diao P, Zhang W, Li J, Ge H, Song Y, Li Z, Wang D, Liu L, Jiang H, Cheng J. 2019.**

Therapeutic Targeting of BRD4 in Head Neck Squamous Cell Carcinoma. *Theranostics***9**:1777–1793 DOI [10.7150/thno.31581](https://doi.org/10.7150/thno.31581).

**Yuanhua L, Pudong Q, Wei Z, Yuan W, Delin L, Yan Z, Geyu L, Bo S. 2019.** TFAP2A Induced KRT16 as an Oncogene in Lung Adenocarcinoma via EMT. *International journal of biological sciences***15**:1419–1428 [DOI 10.7150/ijbs.34076](https://doi.org/10.7150/ijbs.34076).

**Zhang A, Yang J, Ma C, Li F, Luo H. 2021.** Development and Validation of a Robust. Ferroptosis-Related Prognostic Signature in Lung Adenocarcinoma. *Frontiers in cell and developmental biology* **9**:616271 [DOI 10.3389/fcell.2021.616271](https://doi.org/10.3389/fcell.2021.616271).

**Zhang L, Li M, Deng B, Dai N, Feng Y, Shan J, Yang Y, Mao C, Huang P, Xu C, Wang. D. 2019.** HLA-DQB1 expression on tumor cells is a novel favorable prognostic factor for relapse in early-stage lung adenocarcinoma. *Cancer management and research* **11**:2605–2616

[DOI 10.2147/CMAR.S197855](https://doi.org/10.2147/CMAR.S197855).

**Zhang Y, Fan Q, Guo Y, Zhu K. 2020.** Eight-gene signature predicts recurrence in lung adenocarcinoma. *Cancer. biomarkers : section A of Disease markers* **28**:447–457

[DOI 10.3233/CBM-190329](https://doi.org/10.3233/CBM-190329).
